# The Shenzhen Declaration on Plant Sciences – Uniting plant sciences and society to build a green, sustainable Earth

**DOI:** 10.3897/phytokeys.86.20859

**Published:** 2017-09-18

**Authors:** Shenzhen Declaration Drafting Committee

## VITAL CONNECTIONS

Actions and priorities to connect the global community of plant scientists with the world’s changing societies are today more imperative than ever. Environmental degradation, unsustainable resource use, and biodiversity loss all require integrated, collaborative solutions.

## A CHANGING WORLD

As plant scientists we are increasingly aware and concerned with the accelerating rate of change of our planet and our societies. In our lifetimes we have witnessed major alterations in the structure and make-up of land, water, and the atmosphere, in use of natural resources and agricultural practices, in migration of plants, animals, and people, in rates of urbanization, and in the rise and spread of infectious diseases. The rate of species extinction is greater now than at any time in the last 65 million years. It is clear that this tremendous transformation, with its profound effect on nature, is primarily the result of human activities. The degree of pressure on the environment has never been greater — far beyond the level at which natural systems will be able to maintain sustainable productivity. The need to act is urgent.

Equally in transition are our own disciplines in the plant sciences: taxonomy and systematics, morphology and development, evolution and ecology, physiology and genetics. New technologies that generate immense quantities of data are often limited by current infrastructure and information management capabilities; a growing emphasis on laboratory investigations is overshadowing the need for priority field work in rapidly disappearing environments; and balance in training for pure and applied research careers is shifting. In many nations, funding support for basic science is declining along with public trust in science. Parallel to these changes within the plant sciences are those affecting social, political, and economic contexts within which scientific research is conducted. Factors such as growing income inequality among peoples, the uneven redistribution of resources across the globe, and rising levels of conflict within and among nations all impact our ability to conduct meaningful science.

At this time of extraordinary challenges, the International Botanical Congress is being held for the first time in China. The increasing wealth of China and the prosperity of its people, coupled with the country’s need for and interest in tackling serious national environmental problems, have given the country a key role in combatting climate change. China also has the potential to address biodiversity loss through the development and implementation of a strong national plan in this area. The Chinese linking of “risks” with “opportunities” has never carried more meaning than it does now, at a time when all countries need for their own sake, and for the world, to help achieve global sustainability. The hosting of IBC 2017 in Shenzhen, this Declaration, and the establishment of the Shenzhen International Award in Plant Sciences are measures of China’s clear commitment to action.

## THE SHENZHEN CALL FOR ACTION: SEVEN PRIORITIES

We endorse the following seven priorities for strategic action in the plant sciences. Vigorous development of these areas will allow society, with the help of science, to mitigate impacts of human activities on plant species, habitats, and distributions, and to approach formation of a sustainable world for ourselves and those who follow us.

### To become responsible scientists and research communities who pursue plant sciences in the context of a changing world.

Plant scientists must contribute to regional and global sustainability as directly and efficiently as possible. Key efforts, such as the urgent preservation of plant diversity and the adaptation of agriculture to increasingly warm climates, must be strengthened greatly if we are to meet the challenges ahead. Our research is not conducted in a vacuum, and we cannot continue to act as if we have a great deal of time available, when we simply and clearly do not. We must confront challenges swiftly and directly to mitigate rapidly deteriorating environmental conditions.

### To enhance support for the plant sciences to achieve global sustainability.

Plants play a central role in functioning ecosystems. They also are our sole source of food (directly or indirectly), and provide many of our medicines, building materials, clothing materials, and other essential products. Plants deserve a far greater level of scientific attention through enhanced public and private funding than they are receiving at present. Integrated studies are necessary to develop robust solutions to environmental problems. Support across plant sciences, from description to use, should be provided at adequate levels and sustained.

### To cooperate and integrate across nations and regions and to work together across disciplines and cultures to address common goals.

Science is by its very nature international, with the plant sciences no exception. Although progress has been made in moving forward with together, stronger international cooperation will be needed to halt biodiversity loss, improve agriculture, and maintain a stable climate. Working together has never been more important. Stable global partnerships are badly needed to overcome barriers and provide integrated, effective solutions to urgent environmental challenges as rapidly as possible.

### To build and use new technologies and big data platforms to increase exploration and understanding of nature.

New technical approaches to information and information sharing will only accelerate in the years to come, making sustainability of data platforms imperative. Increasingly large, linked databases reveal new connections and relationships about life on Earth. Our rapidly advancing ability to sequence genomes leads to new ways of understanding the diversity, evolution, and functioning of life on our planet. As these and other new technologies expand, we must apply them in timely, integrated, and practical ways to organize information and address environmental problems.

### To accelerate the inventory of life on Earth for the wise use of nature and the benefit of humankind.

More than half of the land plant species could be extinct in nature by the end of the present century. Although we have given names to many, we know very little about most of them, and there are more that await discovery. Those we know now can be protected or preserved, but the urgency of finding and learning about the unknowns before they become extinct is clear. Doing so will require integration and collaboration on a scale we have not yet achieved. We need to know plants in order to save them, but time is short.

### To value, document, and protect indigenous, traditional, and local knowledge about plants and nature.

Indigenous, traditional, and local knowledge about nature is disappearing even more rapidly than is biodiversity itself. Once lost, such knowledge, with its unique insights into nature, can never be regained. Plant scientists must work together with holders of this knowledge to understand and achieve sustainable environmental stewardship. Cultural diversity, coupled with crop genetic diversity, will be of central importance for future food security. We will need informed collaboration coupled with urgent, rigorous planning and implementation across cultures and knowledge systems.

### To engage the power of the public with the power of plants through greater participation and outreach, innovative education, and citizen science.

We need to engage the power of the public with the power of nature. People who care about the environment are motivated to do a great deal to protect it and ensure its future. The creation of an ecological civilization, where societies work together in the creation of knowledge and implementation of solutions, cannot remain only an abstract concept. We all need plants, and plants need our care now more than ever— we depend absolutely upon them for our very existence. Embedding that need into the very fabric of our societies will require global engagement, across nations and cultures — this will require all of us.

We believe that, by working together, we can unite innovative plant sciences with the needs and strengths of human societies, helping to create new paths to a green, sustainable future for Earth, with plants and people in harmony.

### AUTHORS

(Shenzhen Declaration Drafting Committee)

Peter R. Crane^1^, Song Ge^2^, De-Yuan Hong^2^, Hong-Wen Huang^3^, Gen-Lin Jiao^4^, Sandra Knapp^5^, W. John Kress^6^, Harold Mooney^7^, Peter H. Raven^8^, Jun Wen^6^, Wei-Hua Wu^9^, Huan-Ming Yang^10^, Wei-Hua Zhu^11^ and Yu-Xian Zhu^12^

^1^Oak Spring Garden Foundation, 1776 Loughborough Lane, Upperville, VA 20184, USA and Yale School of Forestry & Environmental Studies, New Haven, CT 06511, USA

^2^The State Key Laboratory of Systematic and Evolutionary Botany, Institute of Botany, CAS, Beijing 100093, China

^3^South China Botanical Garden, CAS, Guangzhou, Guangdong 510650, China

^4^Fairy Lake Botanical Garden, Shenzhen & CAS, Shenzhen, Guangdong 518004, China

^5^Department of Life Sciences, Natural History Museum, London SW7 5BD, UK

^6^Department of Botany, National Museum of Natural History, Smithsonian Institution, Washington, DC 20013-7012, USA

^7^Department of Biology, Stanford University, Stanford, CA 94305-5020, USA

^8^Missouri Botanical Garden P.O. Box 299, St. Louis, MO 63166, USA

^9^The State Key Laboratory of Plant Physiology and Biochemistry, China Agricultural University, Beijing 100193, China

^10^BGI, Shenzhen, Guangdong 518083, China

^11^Shenzhen Urban Management Bureau, Shenzhen, Guangdong 518036, China 12 The Institute for Advanced Studies, Wuhan University, Wuhan, Hubei 430072, China

^12^The Institute for Advanced Studies, Wuhan University, Wuhan, Hubei 430072, China

